# The secreted l-arabinose isomerase displays anti-hyperglycemic effects in mice

**DOI:** 10.1186/s12934-015-0391-5

**Published:** 2015-12-21

**Authors:** Moez Rhimi, Luis G. Bermudez-Humaran, Yuan Huang, Samira Boudebbouze, Nadia Gaci, Alexandrine Garnier, Jean-Jacques Gratadoux, Héla Mkaouar, Philippe Langella, Emmanuelle Maguin

**Affiliations:** INRA, UMR 1319 Micalis, Interactions of Firmicutes With Their Environments, 78352 Jouy-en-Josas Cedex, France; AgroParisTech, Micalis, Interactions of Firmicutes With Their Environments, 78352 Jouy-en-Josas Cedex, France; INRA, UMR 1319 Micalis, Commensal and Probiotics-Host Interactions Laboratory, 78352 Jouy-en-Josas Cedex, France; AgroParisTech, Micalis, Commensal and Probiotics-Host Interactions Laboratory, 78352 Jouy-en-Josas Cedex, France

**Keywords:** l-Arabinose isomerase, Secretion, Tagatose, Glycemia, Mice

## Abstract

**Background:**

The l-arabinose isomerase is an intracellular enzyme which converts l-arabinose into l-ribulose in living systems and d-galactose into d-tagatose in industrial processes and at industrial scales. d-tagatose is a natural ketohexose with potential uses in pharmaceutical and food industries. The d-galactose isomerization reaction is thermodynamically equilibrated, and leads to secondary subproducts at high pH. Therefore, an attractive l-arabinose isomerase should be thermoactive and acidotolerant with high catalytic efficiency. While many reports focused on the set out of a low cost process for the industrial production of d-tagatose, these procedures remain costly. When compared to intracellular enzymes, the production of extracellular ones constitutes an interesting strategy to increase the suitability of the biocatalysts.

**Results:**

The l-arabinose isomerase (l-AI) from *Lactobacillus sakei* was expressed in *Lactococcus lactis* in fusion with the signal peptide of *usp45* (SP_*Usp45*_). The l-AI protein and activity were detected only in the supernatant of the induced cultures of the recombinant *L. lactis* demonstrating the secretion in the medium of the intracellular *L. sakei*l-AI in an active form. Moreover, we showed an improvement in the enzyme secretion using either (1) *L. lactis* strains deficient for their two major proteases, ClpP and HtrA, or (2) an enhancer of protein secretion *in L. lactis* fused to the recombinant l-AI with the SP_*Usp45*_. Th l-AI enzyme secreted by the recombinant *L. lactis* strains or produced intracellularly in *E. coli*, showed the same functional properties than the native enzyme. Furthermore, when mice are fed with the *L. lactis* strain secreting the l-AI and galactose, tagatose was produced in vivo and reduced the glycemia index.

**Conclusions:**

We report for the first time the secretion of the intracellular l-arabinose isomerase in the supernatant of food grade *L. lactis* cultures with hardly display other secreted proteins. The secreted l-AI originated from the food grade *L. sakei* 23 K was active and showed the same catalytic and structural properties as the intracellular enzyme. The *L. lactis* strains secreting the l-arabinose isomerase has the ability to produce d-tagatose in vivo and conferred an anti-hyperglycemic effect to mice.

## Background

The l-arabinose isomerase (l-AI, EC 5.3.1.4) is an enzyme that mediates the conversion of l-arabinose to l-ribulose in vivo. At industrial scale this enzyme is used for the conversion of d-galactose into d-tagatose, thus it is also referred to as a d-galactose isomerase [1]. The d-tagatose is a d-fructose isomer currently used as a low calorie sweetener [2]. This ketohexose is a rare natural sugar tasting as sucrose and having similar physical properties [3] although it is not metabolized in humans and consequently has a very low caloric effect [4, 5]. In addition to its sweetener properties, d-tagatose is an anti-hyperglycemic factor and exhibits an efficient anti-biofilm effect. Later, the d-tagatose was considered as a GRAS “Generally Recognized as Safe” sweetener which can be used as a sugar substitute [6].

As previously reported, the isomerization of d-galactose into d-tagatose is thermodynamically equilibrated allowing the shift of the reaction towards the tagatose production when the temperature is increased [6, 7]. Thus, several thermoactive l-AIs have been isolated from thermophilic microorganisms including *Thermotoga*, *Bacillus* and *Thermus* genera [8–10]. However when performed under alkaline conditions, the isomerization has several drawbacks mainly the production of undesirable sub-products [6, 11]. In order to improve the l-AIs suitability for biotechnological applications, many tools have been used such as: the screening of biodiversity to identify relevant enzymes with interesting properties, protein isolation, molecular modeling and rational design [12–14]. In this context, the thermoactivity, the metallic ions requirement and the catalytic efficiency of several enzymes were probed [15, 16]. Recently, new production procedures of d-tagatose were described. We reported the concomitant bioconversion of d-galactose and d-glucose into d-tagatose and d-fructose, respectively. This original procedure was developed through the co-expression of a d-glucose isomerase and an l-arabinose isomerase in *E. coli* [17]. Another study [18] described the hydrolysis of lactose by the β-galactosidase from *Pichia pastoris* to galactose and glucose and the bioconversion of 30 % of d-galactose into d-tagatose with the addition of the l-AI from *Arthrobacter* sp. While several studies revealed that numerous efforts have been made to set out a low cost procedure for the industrial production of tagatose, these processes remain costly [19]. This is mainly due to the biocatalyst production costs. Indeed, the l-AIs are intracellular enzymes that require not only the development of profitable procedures for their over-expression but also for their extraction and purification. Compared to the intracellular enzymes, the production of extracellular biocatalysts is an attractive alternative to improve the industrial process profitability.

Although the proteins belonging to the isomerase family are intracellular, in this study we report for the first time an efficient secretion in the extracellular medium of the l-AI from *Lactobacillus sakei* 23 K by the food grade bacterium *Lactococcus lactis*. We investigated the properties of the secreted protein and its efficacy to bioconvert galactose into tagatose. We also investigated the functionality of the enzyme, the in vivo isomerization of galactose and the cognate anti-hyperglycemic effect of the produced tagatose in mice model.

## Results and discussion

### l-AI secretion in *L. lactis* clpP-htrA strain

l-AI is an intracellular protein, which is an efficient bio-converter of galactose into tagatose. This latter property confers to this enzyme family a strong industrial interest. However, the tagatose production remains to be improved mainly in terms of efficiency and process cost. Many efforts have been focused on the improvement of the biochemical properties of l-AI enzymes and their adaptation to industrial processes. However although the extraction and purification of the intracellular l-AIs increase the cost of production of these biocatalysts, the process steps have hardly been addressed. In this context, we investigated whether an intracellular l-AI could be produced in the extracellular medium. We choose *L. lactis* as the heterologous host for the expression and secretion test because (1) it is a food grade bacterium, (2) it secretes only one detectable extracellular protein (Usp45) [20], (3) it possesses only two major proteases, namely ClpP (intracellular) and HtrA (extracellular) (4) it has been used for the successful expression and secretion of several proteins of medical and industrial interest and for in situ delivery [21].

The gene encoding the l-AI from *L. sakei* 23 K was cloned under the control of p_*nis*_, the nisin-inducible promoter, either directly or in fusion with the signal peptide originated from the *L. lactis usp45* gene. Both constructions as well as the empty vector were transformed in *L. lactis* NZ9000. The activity test showed that the l-AI activity is only found in the Nisin-induced cultures of recombinant *L. lactis* NZ9000 strain and its *clpP*-*htrA* derivative. No activity was observed in the strains harboring the empty plasmid. As expected, l-AI activity was clearly detected (21 ± 0.2 U/mg) in the cell fraction of *L. lactis* NZ9000 strain harboring the pCYT:*araA* plasmid, whereas no signal was detected in the supernatant (Table 1). Similar analysis of *L. lactis* NZ9000 strain harboring the pSEC:*araA* plasmid resulted in an l-AI activity of (10 ± 0.3 U/mg) in the supernatant fraction while not activity was detected in the cell fraction (Table 1). No activity was found in the cellular extracts from the cultures corresponding to the strains harbouring empty vectors. These results demonstrated for the first time that the *L. sakei* 23 K l-AI is expressed in an active form in *L. lactis* not only in its intracellular form but also as a secreted enzyme.

**Table 1:** l-AI specific activity determination in *L. lactis* NZ9000 harboring the secreted and intracellular enzyme forms

Construction	Specific activities (U/mg)	
	Supernatant	Protein crude extract
NZ9000/pCYT:*ara*A	ND	21 ± 0.2
NZ9000/pSEC:*ara*A	10 ± 0.3	ND
NZ9000/pSEC:LEISS:*ara*A	13 ± 0.8	ND

*ND* not detected

**Fig. 1:**
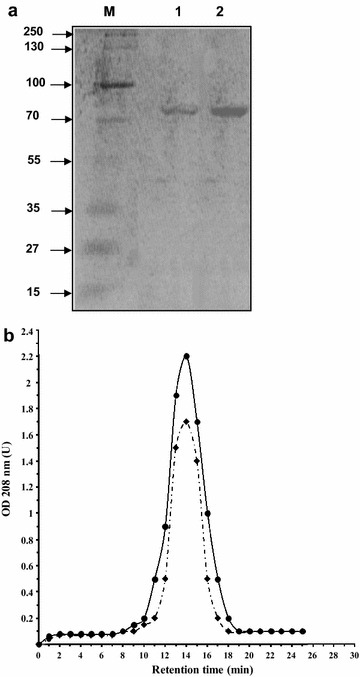
**a** Coomassie brilliant blue-stained gel, under reducing conditions. *Lane 1* protein markers (molecular masses in kilodaltons); *lane 2*, purified secreted l-AI from MRS40; *lane 3*, purified l-AI from *E. coli*. **b** Gel filtration chromatography profiles of the purified l-AI preparation issued from *L. lactis* (*dashed line*) and *E. coli* (*continuous line*). To determine the molecular weight of the purified l-AI preparations we used protein markers of 669 (RT = 8.755 min), 440 (RT = 12.3 min), 232 (RT = 14.02 min), 140 (RT = 16.7 min) and 66 kDa (RT = 20.9 min)

SDS-PAGE analysis of secreted l-AI revealed the presence of a protein band with an apparent molecular weight of about 56 kDa (Fig. 1a). Mass spectrometry experiments confirmed the identity of this band as the l-AI from *L. sakei* 23 K (data not shown). The ability to express the l-AI from *L. sakei* in *L. lactis* is in agreement with a recent report describing the expression of the *Bifidobacterium longum*l-AI in *L. lactis* [18]. However to the best of our knowledge, there is so far no report on the secretion of the l-AI. This work constitutes not only the first report on the secretion of an l-AI, but also an attractive way to produce this protein for biotechnological applications.

To increase the l-AI secretion by *L. lactis* we used the pSEC:LEISS plasmid [22] which contains the *usp45* signal peptide fused to 15 nucleotides that encodes to a 9-residue synthetic propeptide, LEISSTCDA acting as an enhancer of protein secretion in *L. lactis* (Table 2). The l-AI activity was monitored in the supernatants of the *L. lactis* NZ9000 strains harboring the pSEC:*ara*A and pSEC:LEISS:*ara*A plasmids. The l-AI activities measured in the culture supernatants were 10 ± 0.3 and 13 ± 0.8 U/mg for the strains carrying the pSEC:*ara*A and the pSEC:LEISS:*ara*A, respectively i.e. about half of the activity measured in the crude extract of NZ9000 carrying the pCYT:*ara*A vector allowing the intracellular accumulation of the enzyme.

**Table 2:** Bacterial strains and plasmids used in this work

Strains	Genotypes	References
MRS36	*Escherichia coli* BL21 strain harboring the pMR36 plasmid	[30]
NZ9000	MG1363 (*nis*RK genes in chromosome), plasmid free	[33]
NZ9000 *htr*A	NZ9000 defective for the *htr*A gene, plasmid free	[29]
NZ9000 *clp*P	NZ9000 defective for the *clp*P gene, plasmid free	[34]
NZ9000 *htr*A *clp*P	NZ9000 defective for the *htr*A and the *clp*P genes, plasmid free	[35]
MRS37	NZ9000 harbouring the psec:LEISS:*araA*	This work
MRS38	NZ9000 *clp*P harbouring the psec:LEISS:*araA*	This work
MRS39	NZ9000 *htr*A harbouring the psec:LEISS:*araA*	This work
MRS40	NZ9000 *htr*A*clp*P harbouring the psec:LEISS:*araA*	This work

Plasmid	Construct	References
pMR36	Plasmid encoding the l-arabinose isomerase encoding gene (*ara*A) from *L. sakei* 23 K	[30]
pCYT	Vector derived from pGK12 carrying a chloramphenicol resistance gene and the p_nis_ inducible promoter	[22]
pSEC	Vector derived from pGK12 carrying a chloramphenicol resistance gene and the p_nis_ inducible promoter fused to the signal peptide of the *usp45* gene	[22]
pSEC:LEISS	Vector derived from pGK12 carrying a chloramphenicol resistance gene and the p_nis_ inducible promoter in front of the signal peptide of *usp45* fused to a sequence encoding the LEISTCDA polypeptide	[22]
pCYT:*ara*A	pCyt carrying the *L. sakei ara*A gene under the control of the p_nis_ promoter	This work
pSEC:*ara*A	pSec carrying the *L. sakei ara*A gene fused to the signal peptide of *usp45*and under the control of the p_nis_ promoter	This work
pSEC:LEISS:*ara*A	pSec:LEISS carrying under the control of the p_nis_ promoter, the signal peptide of *usp45* associated with the LEISSTCDA encoding fragment and fused to the *L. sakei ara*A gene	This work

It is well known that heterologous proteins can be targeted by the proteases of the expressing host. We investigated the effect of the *L. lactis* proteases on the yield of l-AI production and secretion. Three isogenic mutants of the NZ9000 strain were used; they are deficient in each one or in both proteases of *L. lactis*; the intracellular protease ClpP and/or the extracellular cell surface protease HtrA (Table 2). As shown in Table 3, the measured activity for the *clpP* mutant, the *htrA* mutant and the *clpP*-*htrA* double mutant were 17 ± 0.4, 19 ± 0.3 and 22 ± 0.6 U/mg compared to 13 ± 0.8 U/mg obtained in the wild-type NZ9000 strain carrying the same plasmid. These data indicate that each of the *L. lactis* proteases affects the yield of the secreted l-AI.

**Table 3:** Effect of the *L. lactis* mutant strains on the enzyme secretion efficiency

Strain	Strain designation	Specific activity (U/mg)
NZ9000	MRS37	13 ± 0.8
NZ9000 *clp*P	MRS38	17 ± 0.4
NZ9000 *htr*A	MRS39	19 ± 0.3
NZ9000 *htr*A*clp*P	MRS40	22 ± 0.6

Altogether, the best production levels of the l-AI in *L. lactis* were obtained (1) in the pellet using the pCYT:*ara*A (21 ± 0.2 U/mg) and (2) directly in the supernatant with the combination of the *L. lactis clpP*-*htrA* deficient strain with the pSEC:LEISS:*araA* expression plasmid (22 ± 0.3 U/mg).

### Functional characterization of the secreted l-AI

To study the biochemical properties of the secreted and the intracellular l-AI we purified this protein from *L. lactis* and *E. coli*, respectively. The analysis of the purified protein from *E. coli* and *L. lactis* by SDS-PAGE and gel filtration chromatography showed that both purified enzymes have a tetrameric arrangement (Fig. 1). These results evidenced that the secreted l-AI monomer has a functional tetrameric arrangement in the culture supernatant. This observation is also supported by the fact that the secreted *L. sakei*l-AI and that all l-AIs reported so far, are active as homotetramers or homohexamers [7, 23].

**Fig. 2:**
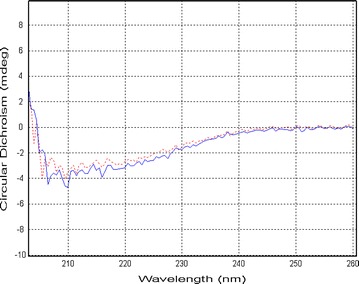
Circular dichroism analysis of the pure l-AI deriving from enzyme by MRS40 *L. lactis* strain (secreted form, in *blue*) and produced in *E. coli* (intracellular form, in *red*)

To go one step further, we analyzed both purified protein fractions by circular dichroism. Our results revealed that the purified proteins from *L. lactis* (secreted form) and *E. coli* (intracellular form) exhibited similar conformations (Fig. 2).

In addition, we studied the activity of the two purified protein preparations as function of the temperature and the pH. Both proteins fractions displayed the same optimal temperature (30–40 °C) and optimal pH (5.0–7.0). The kinetic studies demonstrated that the purified l-AI from *L. lactis* and *E. coli* had a catalytic efficiency of 65 ± 0.8 and 64 ± 0.2/mM/min for l-arabinose, respectively. These results established that the secreted protein not only have the same tetrameric arrangement as the protein over-expressed and purified from *E. coli*, but also the same biochemical and kinetic properties. This underlines again the efficiency of the secretion of this l-AI in *L. lactis* and the relevance of this mode of production for industrial applications. Such procedure constitutes an original promising tool for the secretion of this protein family in this food-grade host microorganism.

### The secreted enzyme efficiently bioconverts d-galactose into d-tagatose

**Fig. 3:**
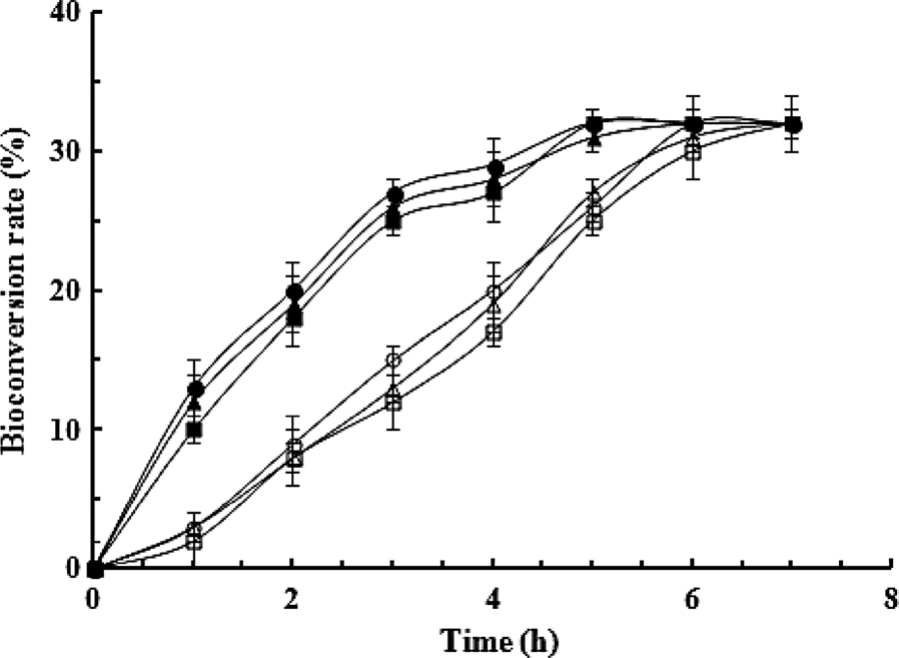
Bioconversion of the d-galactose into d-tagatose at different pH. The conversion was performed by either (1) the induced MRS40 strain: pH 5 (*open circle*), pH 6 (*open triangle*), pH 7 (*open square*) or (2) the purified secreted l-AI enzyme pH 5 (*filled circle*), pH 6 (*filled triangle*), pH 7 (*filled square*)

The ability of the MRS40 strain to bioconvert the d-galactose into d-tagatose was studied. The induced cells are able to produce the d-tagatose from the d-galactose with a production rate of 32 % at pH 5.0, 6.0 and 7.0 (Fig. 3). In contrast in the same conditions, the non-induced MRS40 cells did not produce detectable amount of d-tagatose and the galactose concentration remained stable (data not shown). The purified secreted l-AI displayed a high efficiency to isomerize the d-galactose into d-tagatose (Fig. 3). The analysis of the kinetic of d-galactose isomerization revealed that the bioconversion rates were higher with the purified enzyme (Fig. 3) than with the nisin-induced MRS40 strain. In addition, the highest bioconversion rate was achieved after 5 h in case of the purified enzyme and 7 h with the induced MRS40 cells (Fig. 3). It is likely to be related to a higher concentration of the purified enzyme compared to the amount of l-AI enzyme directly secreted by the induced bacterial culture.

As shown in Fig. 3, the conversion rates were not significantly altered when the pH varied both in case of the induced MRS40 culture and of the purified enzyme. Such phenomenon may be explained by the wide range activity of the *L. sakei*l-AI [24].

These results highlighted the ability of the induced MRS40 cells and of the purified secreted l-AI to biocatalyze the d-tagatose production even at neutral and low pH. This underlines again the efficiency of the l-AI secretion as a powerful system for the over-production of an active extracellular l-AI enzyme.

### The produced d-tagatose in vivo has an antihyperglycemic effect in mice

**Fig. 4:**
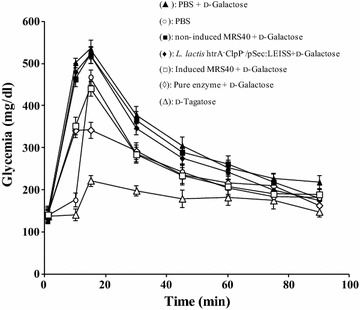
Effect of tagatose on the glycemia in mice. Tests were carried out by administration of tagatose solution or through bioconversion of galactose in vivo using the pure secreted enzyme and the induced MRS40 strain. *Filled triangle* PBS + d-galactose, *filled square* non-induced MRS40, *filled diamond* *L. lactis* htrA^−^ClpP^−^/pSec:LEISS, *open circle* PBS, *open diamond* pure enzyme +d-galactose, *open square* induced MRS40, *open triangle* d-tagatose

The use of tagatose in pharmaceutical applications has been previously reported. One of the main properties of this natural sugar is its anti-hyperglycemic effect [4]. To investigate this property, we studied the impact of the *L. lactis* strain secreting the l-AI and of the purified secreted enzyme on the glycemia of mice receiving d-galactose. This experimental procedure will also evidence the in vivo activity of the l-AI and the ability of the *L. lactis* strain to actively synthesize the l-AI in vivo. As illustrated in Fig. 4, the treatment of mice with d-galactose increased the glycemia values induced by glucose administration when compared to the results obtained with PBS only. On contrast, when the d-glucose administration was preceded by d-tagatose ingestion, the glycemia values did not increased (Fig. 4). These data highlighted the anti-hyperglycemic role of d-tagatose and confirmed what was previously reported regarding the glycemia protecting effect of this natural sugar [4, 25, 26]. We then investigated the efficiency of the secreted l-AI to produce the d-tagatose in vivo and to protect mice against a glycemia increase. As shown in Fig. 4, both non induced MRS40 and *L. lactis clpP htrA*/pSec:LEISS strain did not decreased the mice glycemia values after the administration of glucose. Moreover, the analysis of these glycemia profiles revealed that they were similar to that of the PBS control. The pretreatment of the mice with the nisin-induced MRS40 cells and d-galactose allowed a certain level of protection against the glycemia increase in comparison to the mice treated with PBS and d-galactose. These data strongly suggests that the induced MRS40 strain secreted an active l-AI which was able to bioconvert in vivo the administered galactose into tagatose leading to the glycemia protection typical of this natural sugar. This interpretation is strongly supported by the fact that the mice treated with the purified l-AI enzyme and the d-galactose also showed a significant protection against the induced hyperglycemia (Fig. 4). The decrease of hyperglycemia was the highest after d-tagatose ingestion, intermediate with the ingestion of the purified enzyme and galactose and lower with the ingestion of the induced MRS40 strain and galactose. This observation is in agreement with (1) the elevated amount of d-tagatose used in this experiment (2 mg/mice likely to be in excess), (2) the higher bioconversion activity reached with the purified enzyme compared to that obtained with the induced MRS40 culture as established in our in vitro experiments (Fig. 3). Altogether our results revealed for the first time that the purified and secreted l-AIs convert in vivo the galactose into tagatose as shown by its protective effect against the hyperglycemia observed in the animal model.

## Conclusions

Here we report the secretion of the first l-AI from *L. sakei* 23 K, belonging to the isomerase family, in the food-grade *L. lactis* microorganism. The secreted l-AI from *L. sakei* 23 K displays the same biochemical and structural features when compared to the l-AI produced intracellularly in *E. coli*. Moreover, the *L. lactis* secreted l-AI efficiently bioconverted the d-galactose into d-tagatose in vivo displaying thus an anti-hyperglycemic effect in mice. In the near future we will focus our efforts on the study of the effect of tagatose concentration on the anti-hyperglycemic response. Furthermore, the optimization and/or isolation of new l-AI having high catalytic efficiency and stability will be of interest for the tagatose production at industrial scale. Such features stress the efficiency of the secretion of l-AI as a profitable way to produce active l-AI for industrial applications.

## Methods

### Bacterial strains, media, plasmids and growth conditions

The bacterial strains and plasmids used in this work are listed in Table 2. *E. coli* was grown in Luria–Bertani medium at 37 °C [27]. *L. lactis* was grown in M17 medium supplemented with 1 % glucose at 30 °C without agitation. Plasmids were transformed in *L. lactis* by electroporation [28]. Plasmids were selected by using antibiotics: 5 µg of chloramphenicol per ml for *L. lactis*, 10 µg of chloramphenicol per ml for *E. coli*, and 100 µg of ampicillin per ml for *E. coli*. Nisin was used at final concentration of 10 ng/ml for *L. lactis*. 1 mM of IPTG was used with *E. coli*.

### PCR and DNA manipulation

Preparation of plasmid DNA was performed using the Miniprep kit (Promega). DNA digestion with restriction endonucleases and separation of fragments in agarose gel electrophoresis were performed as described by Sambrook et al. [27]. Polymerase chain reactions were carried out in a Gene Amp PCR System 9700 (Applied Biosystems). The amplification reaction mixtures (100 µl) contained Phusion High-Fidelity DNA polymerase buffer, 10 pmol of each primer, 50 ng of DNA template, and 10 units of High-Fidelity DNA polymerase (Fermentas). The cycling parameters were 94 °C for 5 min, followed by 35 cycles at 94 °C for 30 s, 55 °C for 30 s and 72 °C for 90 s and finally 7 min at 72 °C. PCR products were purified using the GFX™ PCR DNA and Gel Band Purification Kit (Amersham Bioscience), following the manufacturer’s instructions.

### l-Arabinose isomerase molecular cloning strategy

The pMR36 plasmid was used as a template for the amplification of the gene encoding the l-AI from *Lactobacillus sakei* 23 K (Table 2). Two primers F-araA: AACTGCAGCATTAAATACAGAAAATTATGAATTTTGG and R-araA: GGACTAGTCCTTATTTAATATTGACGTAAGTCAAATC were used to amplify the *araA* gene flanked by the *Spe*I and *Pst*I restriction sites. The resulting PCR fragment was purified and then digested with the latter restriction enzymes. The digested fragment was purified and subsequently ligated to the pCYT, pSEC and pSEC:LEISS vectors (to obtain pCYT:*ara*A, pSEC:*ara*A and pSEC:LEISS:*ara*A, respectively) linearized with *Spe*I and *Nsi*I restriction enzymes (Table 2). The ligation products were then transformed into either *L. lactis* NZ9000 or *L. lactis clpP*-*htrA* strain [29]. Recombinant clones were analyzed by restriction and generated constructions were confirmed by DNA sequencing using an automated DNA sequencer (MWG Eurofins).

### Preparation of crude extracts and protein purification

Recombinant *L. lactis* were grown until OD_600_ = 0.6 and induction with 10 ng/ml of nisin (Sigma) was performed during 3 h. The cells were harvested and the supernatant was concentrated using a 100 kDa cut-off membrane. The resulting protein fraction was subjected to an anion exchange chromatography (Mono-Q 5/50GL, GE Healthcare). Purification was achieved by a size exclusion chromatography step (S200 column, Amersham Bioscience) using an ÄKTA purifier system (Amersham Biosciences). The used buffer was 100 mM sodium acetate (pH 5.0) and elution fractions were 0.5 ml.

The harvested *L. lactis* cells were washed twice with 100 mM sodium acetate buffer (pH 5.0) and disrupted by glass beads (diameter of 212–300 µm, v/v, Sigma). Crude cell extract were recovered by centrifugation (30,000×*g*, 20 min at 4 °C).

*Lactobacillus sakei*l-AI produced in *E. coli* strain (MRS36) was over-expressed and purified as previously reported [30].

### Protein quantification and electrophoresis

Protein concentrations were determined using the Bradford method with bovine serum albumin as standard. The purified enzyme samples were migrated in 12 % sodium dodecyl sulfate–polyacrylamide gel electrophoresis (SDS-PAGE) according to the method of Laemmli [31]. Protein bands were visualized by Coomassie brilliant blue R-250 (BioRad) staining.

### Enzyme assays

l-AI activity was established by determining the amount of generated l-ribulose or d-tagatose. Under standard conditions, the reaction mixture contained 0.8 mM Mg^2+^, 0.8 mM Mn^2+^, 50 μl of enzyme preparation at a suitable dilution; 5 mM of l-arabinose (or d-galactose) and sodium acetate buffer 100 mM (pH 5.0) to bring the final volume to 1 ml. The reaction mixture was incubated at 35 °C during 1 or 10 min for l-arabinose and d-galactose, respectively, followed by the incubation of the samples at 99 °C during 5 min. The generated l-ribulose (or d-tagatose) was measured by the cysteine carbazole sulfuric-acid method, and the absorbance was measured at 560 nm [32]. The d-tagatose production was also confirmed by high-pressure liquid chromatography (HPLC).

One unit of l-AI activity was defined as the amount of enzyme catalyzing the formation of 1 µmol keto-sugar per min under the above-specified conditions.

### Circular dichroism experiments

Circular dichroism (CD) measurements were done with chirascan spectropolarimeter (Applied photophysics). Purified proteins were used at concentration of 1 mg/ml. The CD spectra of enzyme samples in a cuvette (0.1 cm) path length were analyzed in the far-UV region comprised between 200 and 280 nm. Scans were collected at 0.1 nm intervals with a 1 nm bandwidth five times. Each spectrum was corrected by subtracting that of the solution containing the used buffer.

### Biochemical and kinetic characterization

The effect of temperature on the activity was determined by incubating the purified enzyme at temperatures ranging from 4 to 55 °C, whereas the pH profile was obtained by measuring the activity at various pH values from 3.0 to 8.5 [3.0–5.0 with sodium acetate buffer, 6.0–7.0 with 2-morpholinoethanesulfonic acid (MES) buffer and 7.5–8.5 with Bicine buffer].

Kinetic properties were studied on the basis of Lineweaver–Burk plots. Assays were done in 100 mM sodium acetate buffer (pH 5.0), 0.8 mM Mg^2+^, 0.8 mM Mn^2+^ and 1–800 mM substrate (l-arabinose or d-galactose). Samples were incubated at 35 °C and the amount of keto-sugar generated (l-ribulose or d-tagatose) was determined by the cysteine-carbazole-sulfuric acid method [32].

### In vitro d-galactose bioconversion

Induced MRS40 cells and purified secreted l-AI with a final concentration of 10^9^ CFU/ml and 0.3 mg/ml, respectively, were used to produce d-tagatose. Reactions were carried out under different pH values (pH 5.0, 6.0 and 7.0) by using 10 g/l d-galactose. d-tagatose was determined by cysteine carbazole method [32].

### Animal experiments and glycemia measurement

Male C57BL/6 mice (6–8 weeks old) (Janvier, Le Genest 428 Saint Isle, France or Taconic mice New York, USA) were maintained at the animal care facilities of the National Institute of Agricultural Research (IERP, INRA, Jouy-en-Josas, France) under specific pathogen-free conditions. Mice were housed under standard conditions for a minimum of 1 week before experimentation. All experiments were performed in accordance with European Community rules and approved by the Animal Care Committee COMETHEA (Comité d’Ethique en Expérimentation Animale du Centre INRA de Jouy-en-Josas et AgroParisTech, Jouy en Josas, France). Food intake was stopped 6 h before starting experiments.

*L. lactis* cellular pellets were harvested by centrifugation (3000*g*, at 4 °C) and washed three times with sterile PBS. The pellet was suspended in PBS to a final concentration of 10^9^ CFU. Groups of mice (*n* = 10) received a intragastric administration of either PBS, d-galactose (10 g/l), d-tagatose (10 g/l) or d-galactose (10 g/l) with: (1) MRS40 strain carrying the pSEC:LEISS vector, (2) induced MRS40 strain carrying the pSEC:LEISS:*ara*A vector, (3) non-induced MRS40 strain carrying the pSEC:LEISS:*ara*A vector and (4) the purified enzyme (0.3 mg/ml). The administered volume was 0.2 ml for each condition followed by a glucose challenge (1 g/kg body weight). Glycemia measurements were done by using the Accu-Chek performa system (Roche).

### Statistical analysis

The data reported in this work were plotted using Sigma Plot (Version 9.0). Each value represents the mean for three independent experiments performed in duplicate.
